# RETRACTED: Umakanthan et al. COVID-19 Vaccine Hesitancy and Resistance in India Explored through a Population-Based Longitudinal Survey. *Vaccines* 2021, *9*, 1064

**DOI:** 10.3390/vaccines13050499

**Published:** 2025-05-08

**Authors:** Srikanth Umakanthan, Sonal Patil, Naveen Subramaniam, Ria Sharma

**Affiliations:** 1 Department of Para-Clinical Sciences, Faculty of Medical Sciences, The University of the West Indies, St Augustine, Trinidad and Tobago; 2 Department of Community Medicine, RRN Hospital and Research Center, Madurai 625501, Tamil Nadu, India; sonaldrpatil@gmail.com (S.P.); naveen.subbu.rrn@gmail.com (N.S.); 3 Medical Resident, RRN Hospital and Research Center, Madurai 625501, Tamil Nadu, India; ria.sharma.dr@gmail.com

The journal retracts the article, titled “COVID-19 Vaccine Hesitancy and Resistance in India Explored through a Population-Based Longitudinal Survey” [1], cited above.

Following publication, concerns were brought to the attention of the Editorial Office regarding an overlap between this publication [1] and a previously published article [2] produced by a different authorship group.

Adhering to our complaints procedure, the Editorial Office and Editorial Board conducted an investigation that confirmed a significant overlap between these two articles [1,2], without the appropriate acknowledgment or citation. Given the extent of the overlap, the Editorial Board considers this publication to be redundant and have decided to retract this paper as per MDPI’s retraction policy (https://www.mdpi.com/ethics#_bookmark30).

This retraction was approved by the Editor-in Chief of the *Vaccines* journal.

The authors did not agree to this retraction.
